# The socioeconomic burden of chronic lung disease in low-resource settings across the globe – an observational FRESH AIR study

**DOI:** 10.1186/s12931-019-1255-z

**Published:** 2019-12-21

**Authors:** Evelyn A. Brakema, Aizhamal Tabyshova, Rianne M. J. J. van der Kleij, Talant Sooronbaev, Christos Lionis, Marilena Anastasaki, Pham Le An, Luan Than Nguyen, Bruce Kirenga, Simon Walusimbi, Maarten J. Postma, Niels H. Chavannes, Job F. M. van Boven, On behalf of the FRESH AIR collaborators, Pham Le An, Marilena Anastasaki, Azamat Akylbekov, Andy Barton, Antonios Bertsias, Pham Duong Uyen Binh, Job F. M. van Boven, Evelyn A. Brakema, Dennis Burges, Lucy Cartwright, Vasiliki E. Chatzea, Niels H. Chavannes, Liza Cragg, Tran Ngoc Dang, Ilyas Dautov, Berik Emilov, Irene Ferarrio, Frederik A. van Gemert, Ben Hedrick, Le Huynh Thi Cam Hong, Nick Hopkinson, Elvira Isaeva, Rupert Jones, Corina de Jong, Sanne van Kampen, Winceslaus Katagira, Bruce Kirenga, Jesper Kjærgaard, Rianne M. J. J. van der Kleij, Janwillem Kocks, Le Thi Tuyet Lan, Tran Thanh Duv Linh, Christos Lionis, Kim Xuan Loan, Maamed Mademilov, Andy McEwen, Patrick Musinguzi, Rebecca Nantanda, Grace Ndeezi, Sophia Papadakis, Hilary Pinnock, Jillian Pooler, Charlotte C. Poot, Maarten J. Postma, Anja Poulsen, Pippa Powell, Nguyen Nhat Quynh, Susanne Reventlow, Dimitra Sifaki-Pistolla, Sally Singh, Talant Sooronbaev, Jaime Correia de Sousa, James Stout, Marianne Stubbe Østergaard, Aizhamal Tabyshova, Ioanna Tsiligianni, Tran Diep Tuan, James Tumwine, Le Thanh Van, Nguyen Nhu Vinh, Simon Walusimbi, Louise Warren, Sian Williams

**Affiliations:** 1 Department of Public Health and Primary Care, Leiden University Medical Center, Postzone V0-P, Postbus 9600, 2300 RC Leiden, The Netherlands; 2 Pulmonary Department, National Center of Cardiology and Internal Medicine, Bishkek, Kyrgyzstan; 3 Unit of Global Health, Department of Health Sciences, University of Groningen, University Medical Center Groningen, Groningen, The Netherlands; 4 Clinic of Social and Family Medicine, School of Medicine, University of Crete, Heraklion, Crete Greece; 5 University of Medicine and Pharmacy, Ho Chi Minh City, Vietnam; 6 Department of Medicine and Makerere Lung Institute, Makerere University, Kampala, Uganda; 7 Department of General Practice & Elderly Care Medicine, University of Groningen, University Medical Center Groningen, Groningen Research Institute for Asthma and COPD (GRIAC), Groningen, The Netherlands

**Keywords:** Chronic respiratory disease, Chronic lung disease, Obstructive lung disease, WPAI, Health economics, Low-income population, Work, Low-resource countries, Household air pollution

## Abstract

**Background:**

Low-resource settings are disproportionally burdened by chronic lung disease due to early childhood disadvantages and indoor/outdoor air pollution. However, data on the socioeconomic impact of respiratory diseases in these settings are largely lacking. Therefore, we aimed to estimate the chronic lung disease-related socioeconomic burden in diverse low-resource settings across the globe. To inform governmental and health policy, we focused on work productivity and activity impairment and its modifiable clinical and environmental risk factors.

**Methods:**

We performed a cross-sectional, observational FRESH AIR study in Uganda, Vietnam, Kyrgyzstan, and Greece. We assessed the chronic lung disease-related socioeconomic burden using validated questionnaires among spirometry-diagnosed COPD and/or asthma patients (total *N* = 1040). Predictors for a higher burden were studied using multivariable linear regression models including demographics (e.g. age, gender), health parameters (breathlessness, comorbidities), and risk factors for chronic lung disease (smoking, solid fuel use). We applied identical models per country, which we subsequently meta-analyzed.

**Results:**

Employed patients reported a median [IQR] overall work impairment due to chronic lung disease of 30% [1.8–51.7] and decreased productivity (presenteeism) of 20.0% [0.0–40.0]. Remarkably, work time missed (absenteeism) was 0.0% [0.0–16.7]. The total population reported 40.0% [20.0–60.0] impairment in daily activities. Breathlessness severity (MRC-scale) (B = 8.92, 95%CI = 7.47–10.36), smoking (B = 5.97, 95%CI = 1.73–10.22), and solid fuel use (B = 3.94, 95%CI = 0.56–7.31) were potentially modifiable risk factors for impairment.

**Conclusions:**

In low-resource settings, chronic lung disease-related absenteeism is relatively low compared to the substantial presenteeism and activity impairment. Possibly, given the lack of social security systems, relatively few people take days off work at the expense of decreased productivity. Breathlessness (MRC-score), smoking, and solid fuel use are potentially modifiable predictors for higher impairment. Results warrant increased awareness, preventive actions and clinical management of lung diseases in low-resource settings from health policymakers and healthcare workers.

## Background

Low- and middle-income countries account for more than 90% of the global COPD mortality and 80% of the asthma mortality [1]. Also regarding the socioeconomic burden, low-resource settings seem disproportionally affected [2–4]. In these settings, increased predisposition to chronic lung diseases (CLDs) already starts in-utero due to high exposure to environmental risk factors (such as excessive indoor and outdoor air pollution) and poorer living conditions (e.g. undernutrition) [3, 5–12]. Hence CLDs develop in a younger, primarily working-age, population [5, 13–15]. Furthermore, the patient burden is particularly high in low-resource settings, because CLDs manifest themselves more severely due to suboptimal (access to) care, including diagnostic- and treatment options [2–4, 16]. Severe CLDs can impact patients’ daily activities substantially [17]. With often limited or non-existent social security systems, families are left in severe trouble when their breadwinner can no longer support them financially [2, 18]. Paradoxically, most studies on the CLD-associated socioeconomic burden have been performed in high-resource settings. The urgency of evaluating outcomes specifically in low-resource settings was therefore underlined recently [19]. In particular, the need for social, economic, and policy research was highlighted as crucial for diminishing the burden of CLD in LMICs [12].

An important form of the social burden of CLD is impairment of patients’ daily activities [17]. On top of that comes the direct economic burden (such as medication and hospital visits) and indirect economic burden (such as productivity loss at work) [20, 21]. While widely available in high income countries, data on the social and indirect economic burden in low-resource settings remain especially scarce [2, 22, 23]. One study reported on an indirect burden, unemployment, for both high- and low-resource settings [24]. It observed a relation between chronic airflow obstruction and unemployment only for high-resource settings. However, as employment was a dichotomized outcome, disease-related hours missed from work (absenteeism) were not taken into account. In addition, while being at work, symptoms of CLD can seriously impact productivity (presenteeism). Presenteeism is more responsive to asthma control than absenteeism and is a vital source of preventable burden [20]. Hence, the actual socioeconomic impact of CLDs in low-resource settings has yet to be uncovered.

Gaining more knowledge on the actual socioeconomic burden is of critical importance to adequately inform policymakers, healthcare professionals, and community members on the impact of CLDs [12]. Evidence on the burden can raise awareness and encourage prioritization of the use of scarcely available resources for CLDs, so that these can be approached with highly (cost-)effective interventions [2, 3]. Furthermore, there is a need to identify (modifiable) risk factors for impairment [25], which may allow targeted interventions. Therefore, the aim of this study was to estimate the socioeconomic burden of CLD in diverse low-resource settings across the globe. To inform governmental and public health policy, we focused on work productivity and activity impairment and its modifiable clinical and environmental risk factors.

## Methods

This study was part of the FRESH AIR project (Free Respiratory Evaluation and Smoke-exposure reduction by primary Health cAre Integrated gRoups; trial registration number: NTR5759), targeting (implementation of) the prevention, diagnosis, and treatment of CLDs in low-resource settings [26]. An online supplement provides additional information on the methods (Additional file 1: Appendix 2).

### Design and setting

This observational, cross-sectional study was performed between July 2016 and March 2018 in Uganda, Vietnam, Kyrgyzstan, and rural Greece. The study sites were sampled purposefully to represent four distinct low-resource settings in terms of geography, ethnicity, risk factor exposure, and healthcare- and political system. At these sites, we selected healthcare centers routinely using spirometry to diagnose CLDs (asthma, COPD, or asthma-COPD overlap (ACO)). The exact selection method of settings and participants was designed in close collaboration with the local teams to meet their daily clinical routine, typical patient population, and available resources (Additional file 1: Appendix 2; Table E1).

### Participants

We recruited participants consecutively during visits to the selected health centers (Additional file 1: Appendix 2 Table E1). We included patients ≥15 years with a spirometry-confirmed diagnosis of COPD [27], asthma or ACO [28]. We did not deploy additional inclusion criteria for COPD (age, tobacco use), as patients in low-resource settings may develop COPD earlier in life due to disadvantage factors such as household air pollution [5, 6, 10, 13]. Patients with a disability hampering communication, too severely ill to participate, or with missing outcomes on activity impairment, were excluded.

### Procedures

Eligible participants were identified and informed about the study by their physicians during a routine visit. After consent, participants filled out a questionnaire. Their physician added the clinical data from existing medical history files. In three hospitals in Kyrgyzstan, well-organized patient registries allowed research-assistants to recruit participants per telephone to administer the questionnaire (Additional file 1: Appendix 2 provides further details on the procedures).

### Instruments

The questionnaire was composed of several validated [29, 30], structured questionnaires with additional open-ended questions, assessing demographic, socioeconomic, and health factors (Additional file 1: Appendix 3). The outcome work- and other activity impairment was assessed using the recommended Work Productivity- and Activity Impairment (WPAI) questionnaire [30–32]. The WPAI-questionnaire assesses CLD -related absenteeism, presenteeism, overall work impairment (absenteeism and presenteeism combined), and impairment of regular activities during the preceding 7 days [30]. All items are calculated into percentages (Additional file 1: Appendix 3), with higher numbers indicating greater impairment and less productivity. When available, we used official, validated WPAI-translations [33].

All questions were asked in the local language (English, Vietnamese, Russian, Greek). In Uganda, where several local languages are spoken, the involved research-team represented all major language groups. We piloted the questionnaire and improved the translation and contextual adaptations accordingly. For example, as many patients were unaware of the name of their disease, we added clarifications on CLDs before asking about the impact of their ‘COPD’ and/or ‘asthma’.

### Sample size

With a total covered population of +/− 146 million (Uganda: 40; Kyrgyzstan: 6; Vietnam: 90; Greece: 10 million), an estimated global CLD-prevalence of 5% [22, 34], a number of 1040 participants resulted in a 99% confidence level and a 4% error margin. Notably, CLD-prevalence is mostly unknown in our diverse low-resource settings. Therefore, the sample size was not calculated to compare between countries and not weighted based on country-size or differences in prevalence.

### Statistical analysis

Population characteristics and the WPAI were analyzed using descriptive statistics (SPSS version 25; IBM, Armonk, NY, USA). The relation between predictors and activity impairment was first assessed per country, using univariable and forced-entry multivariable linear regressions. An identical regression model was used for each country, based on known risk factors for impairment (Additional file 1: Appendix 2) [35–37]. We added solid fuel use for cooking/heating, as besides smoking this is another major risk factor for CLD in low-resource settings [1, 8, 10, 38]. There were no indications for multicollinearity. The unstandardized coefficients of each country with their 95% confidence intervals (CI) were then meta-analyzed (Comprehensive Meta-Analysis version 3; Biostat, Englewood, NJ, USA). We generally used a fixed-model. Only for ‘comorbidity’ we used a random-effect model, as for this variable there were indications for heterogeneity of effect between the countries. Because our Kyrgyz population had no asthma patients, we performed a separate meta-analysis without this country to check for any differences (Additional file 1: Appendix 4 Table E4). Coefficients with 95%CI excluding 1 were considered statistically significant.

## Results

### Clinical and demographic characteristics

**Fig. 1:**
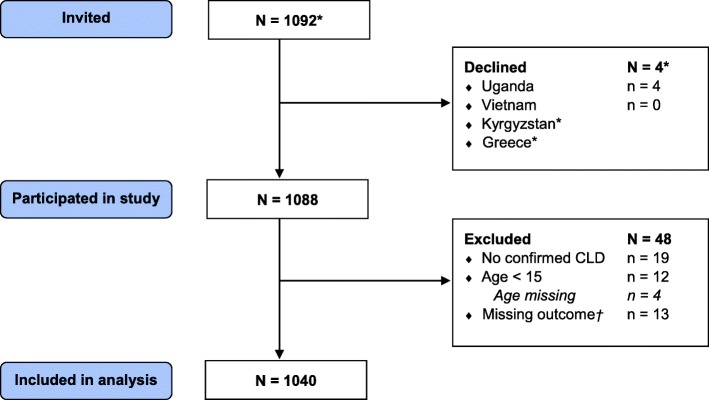
Recruitment of study participants. CLD = chronic lung disease. *In Greece and Kyrgyzstan, the exact number was not registered during the process. †Participants were excluded from the analysis if the outcome ‘activity impairment’ was missing

We included a total of 1040 participants (Fig. 1); most were recruited in Vietnam and Kyrgyzstan. Almost half of the total population was male, and the median age was 60.0 [IQR 48.0–70.0] (Table 1). The Ugandan population consisted of more female and younger participants, whereas the other countries’ populations consisted of somewhat more male participants of older age. A slight majority of the participants was diagnosed with COPD (55.1%), followed by asthma (38.5%), and a small number with ACO (6.4%). Breathlessness severity was generally moderate (median MRC-score 3.0; IQR 2.0–4.0). Having at least one comorbidity was common (34.7%), with heart disease being most prevalent (Additional file 1: Appendix 4 Table E1). Risk factors for developing CLD were highly prevalent, such as having ever smoked daily (43.9% of whom 91.7% male), solid fuel use (54.0%) and occupational exposure to dust or fumes (59.4% of the 401 workers). The distribution of risk factors differed across the countries. For example, in Uganda, smoking prevalence was extremely low (3.5%) compared to solid fuel use (98.8%), whereas in Greece this was the other way around (68.9 and 49.4% respectively). Clinical and demographic details are reported in Additional file 1: Appendix 4 Table E1.

**Table 1:** Clinical and demographic characteristics

	Uganda *N* = 173 (16.6%)	Vietnam *N* = 471 (45.3%)	Kyrgyzstan *N* = 306 (29.4%)	Greece *N* = 90 (8.7%)	Total *N* = 1040 (100%)
Demographic characteristics					
Male	39 (22.5)	274 (58.2)	188 (61.4)	55 (61.1)	556 (53.3)
Age (yrs.), median [IQR]	35.0 [22.5–47.0]	62.0 [52.0–72.0]	62.0 [55.0–70.0]	72.0 [63.8–79.0]	60.0 [48.0–70.0]
BMI (kg/m^2^), median [IQR]	23.8 [20.4–28.3]	21.9 [19.5–24.4]	25.8 [23.7–29.4]	28.0 [24.7–31.5]	23.9 [20.8–27.3]
Higher education^a^	46 (26.7)	156 (33.1)	291 (95.1)	4 (4.4)	497 (47.8)
Working status					
Working	93 (53.8)	193 (41.1)	92 (30.1)	23 (25.6)	401 (38.6)
*Employed (for payment)*	*81 (87.1)*	*134 (69.4)*	*40 (43.5)*	*15 (65.2)*	*270 (67.3)*
Not working	41 (23.7)	153 (32.6)	34 (11.1)	13 (14.4)	241 (23.2)
Student	36 (20.0)	5 (1.1)	0 (0.0)	0 (0.0)	41 (3.9)
Retired	3 (1.7)	119 (25.3)	180 (58.8)	54 (60.0)	356 (34.2)
Having child (ren)	117 (67.6)	417 (88.5)	302 (98.7)	79 (87.8)	915 (88.0)
Ever smoker	6 (3.5)	251 (53.3)	138 (45.1)	62 (68.9)	457 (43.9)
*Pack years, median [IQR]*	*3.8 [2.0–19.9]*	*29.0 [15.5–44.0]*	*27.0 [14.2–40.8]*	*57.0 [26.1–74.0]*	*30.0 [15.1–45.0]*
*Male*	*4 (66.7)*	*234 (93.2)*	*134 (97.1)*	*47 (75.8)*	*419 (91.7)*
*Current smoker*	*6 (100.0)*	*92 (36.7)*	*37 (26.8)*	*40 (64.5)*	*175 (38.3)*
Solid fuel use	170 (98.8)	130 (27.6)	218 (71.5)	44 (49.4)	562 (54.0)
Occupational exposure^b^	87 (93.5)	104 (53.9)	37 (40.2)	10 (43.5)	238 (59.4)
Health characteristics					
Diagnosed as:					
COPD	11 (6.4)	190 (40.3)	305 (99.7)	67 (74.4)	573 (55.1)
Asthma	161 (93.1)	223 (47.3)	0 (0.0)	16 (17.8)	400 (38.5)
ACO	1 (0.6)	58 (12.3)	1 (0.3)	7 (7.8)	67 (6.4)
Breathlessness severity (MRC-scale), median [IQR]	2.0 [1.0–2.0]	3.0 [2.0–4.0]	4.0 [3.0–4.0]	2.0 [2.0–4.0]	3.0 [2.0–4.0]
Exacerbation(s) in past year	0 (0.0)	102 (21.7)	35 (11.4)	9 (10.0)	146 (14.0)
Comorbidity (any)	27 (15.6)	228 (48.4)	62 (20.3)	44 (48.9)	361 (34.7)

Data are in numbers (%) unless stated otherwise. *ACO* asthma-COPD overlap, *BMI* body mass index, *IQR* interquartile range, *MRC* medical research council. Text in italics means category within category above. ^a^ Above secondary education. ^b^Regards only those working. Missing values N (%) for BMI 6 (0.6) in G; education 1 (0.1) in U; working status 1 (0.1) in V; pack years 13 (1.2) 1 in G, 2 in V, 10 in K; solid fuel use 3 (0.3) 1 in U, K, and G; MRC-score 1 (0.1) in U; exacerbation 1 (0.1) in G

### Work productivity and activity impairment

**Fig. 2:**
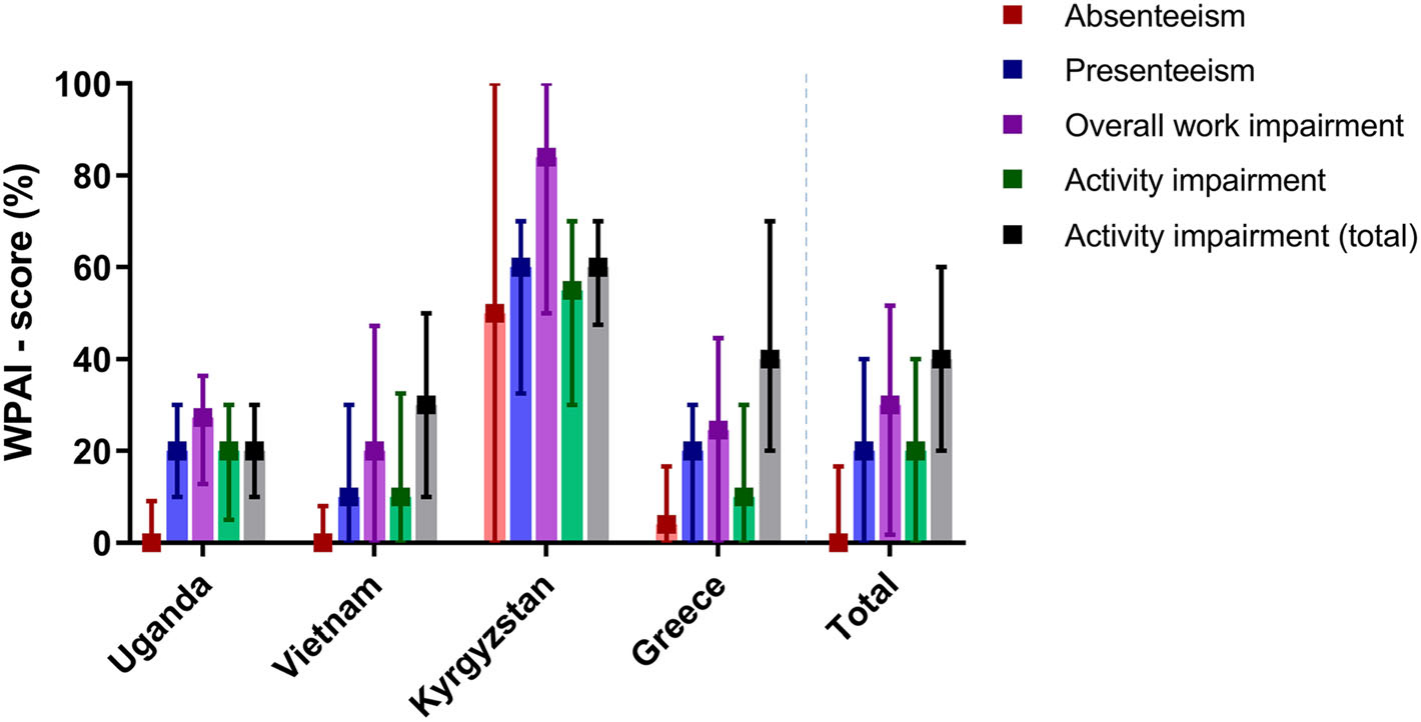
Work productivity and activity impairment due to CLD. CLD = chronic lung disease; WPAI = work productivity and activity impairment in median [interquartile range] %. 100% means maximum impairment. Total number of participants (numbers of employed population): Uganda *N* = 173 (81), Vietnam 471 (134), Kyrgyzstan 306 (40), Greece 90 (15), and total 1040 (270). Due to different population characteristics per country, data should be interpreted within the country’s context and not be used to directly compare between countries

Locally, 533 participants (51.2%) were considered to be of working age (Additional file 1: Appendix 4 Table E1). Although 401 identified themselves as ‘working’, 270 (67.3%) of those worked for a salary at an employer. WPAI-scores were generally very similar across the countries. However, in Kyrgyzstan, all scores were higher (Fig. 2, Table 2). Still, a similar pattern was visible in each country: while CLD-related absenteeism was (relatively) very low among those employed, presenteeism was relatively high, leading to a substantial overall work impairment. Activity impairment was considerably high, particularly in the total population. To facilitate interpretation of the outcomes within their country, WPAI-scores and their 95% CIs are provided in Additional file 1: Appendix 4 Table E2.

**Table 2:** CLD-related work productivity and activity impairment (WPAI)

WPAI item	Uganda	Vietnam	Kyrgyzstan	Greece	Total
Employed population					
Absenteeism					
% work time missed due to CLD	0.0 [0.0–9.1]	0.0 [0.0–8.0]	50.0 [0.0–100.0]	4.0 [0.0–16.7]	0.0 [0.0–16.7]
% of the people who missed any work due to CLD, mean (95%CI)	46.8 (35.6–58.1)	31.5 (23.3–39.7)	70.0 (55.2–84.8)	50.0 (20.0–80.0)	43.0 (36.9–49.1)
Presenteeism					
% impairment while working due to CLD	20.0 [10.0–30.0]	10.0 [0.0–30.0]	60.0 [32.5–70.0]	20.0 [0.0–30.0]	20.0 [0.0–40.0]
% of the people whose productivity was affected, mean (95%CI)	77.2 (67.8–86.7)	62.2 (53.7–70.8)	100.0 (100.0–100.0)	71.4 (44.4–98.5)	72.9 (67.4–78.3)
Overall work impairment					
Absenteeism and presenteeism combined	27.3 [12.9–36.7]	20.0 [0.0–47.3]	84.0 [50.0–100.0]	24.5 [0.0–44.6]	30.0 [1.8–51.7]
% of people who suffered from any work impairment, mean (95%CI)	79.7 (70.7–88.8)	65.4 (57.0–73.7)	100.0 (100.0–100.0)	71.4 (44.4–98.5)	75.2 (69.9–80.5)
Activity impairment					
% impairment of activities due to CLD	20.0 [5.0–30.0]	10.0 [0.0–32.5]	55.0 [30.0–70.0]	10.0 [0.0–30.0]	20.0 [0.0–40.0]
% of the people whose daily activities were affected, mean (95%CI)	75.9 (66.3–85.6)	61.4 (52.8–70.0)	100.0 (100.0–100.0)	53.8 (22.5–85.2)	71.5 (66.1–76.9)
Total population					
Activity impairment					
% impairment of activities due to CLD	20.0 [10.0–30.0]	30.0 [10.0–50.0]	60.0 [47.5–70.0]	40.0 [20.0–70.0]	40.0 [20.0–60.0]
% of the people whose daily activities were affected, mean (95%CI)	80.3 (74.4–86.3)	80.5 (76.9–84.1)	98.0 (96.5–99.6)	90.0 (83.7–96.3)	86.4 (84.4–88.5)

Data are in median [interquartile range] unless stated otherwise. *CI* confidence interval; *CLD* chronic lung disease. Total number of participants (numbers of employed population): Uganda *N* = 173 (81), Vietnam 471 (134), Kyrgyzstan 306 (40), Greece 90 (15), and total 1040 (270). Due to different population characteristics per country, data should be interpreted within the country’s context and not be used to directly compare between countries

The proportion of patients that suffered from any degree of impairment due to their CLD during the past seven days was also high for all four WPAI outcomes. Although many patients missed any amount of work time (43.0%), the work time they missed was very low. On the contrary, the proportion of patients who suffered from activity impairment was much higher (86.4%) and also the level of activity impairment was high.

### Risk factors for activity impairment

**Fig. 3:**
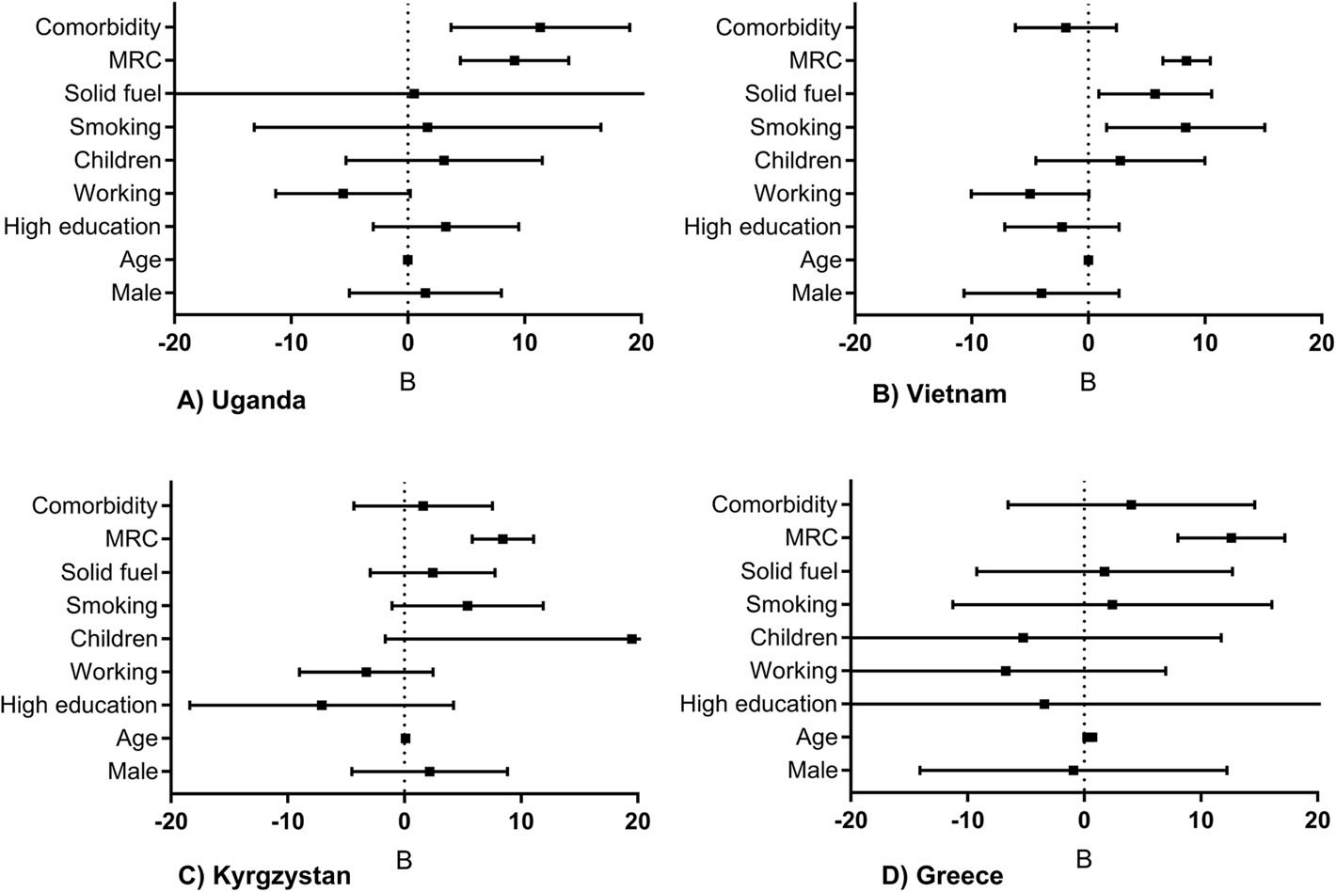
Multivariable regressions per country. Mean unstandardized B (95%CI). MRC = medical research council breathlessness scale (ranging 1-5). Age (years). **a** Uganda, **b** Vietnam, **c** Kyrgyzstan, **d** Greece

**Fig. 4:**
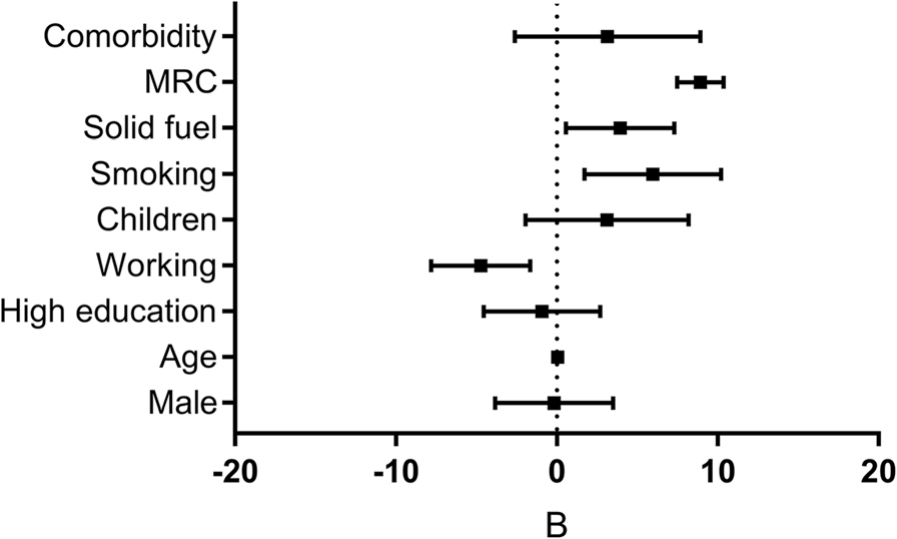
Total multivariable regression. Mean unstandardized B (95%CI). MRC = medical research council breathlessness scale (1-5). Age (years)

**Fig. 5:**
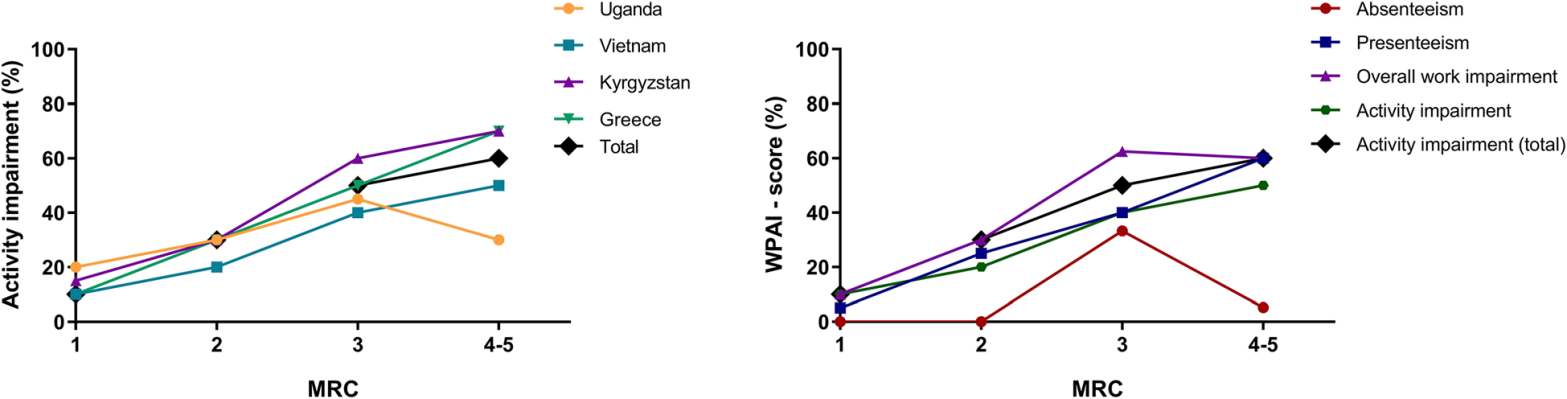
WPAI and MRC-score. MRC = medical research council breathlessness scale (ranging 1-5). WPAI = work productivity and activity impairment in median %. Left: Activity impairment per MRC-score per country; Uganda *N* = 172 (1 missing MRC value), Vietnam *N* = 471, Kyrgyzstan *N* = 306, Greece *N* = 90, and total *N* = 10. Due to different population characteristics per country, data should be interpreted within the country’s context and not be used to directly compare between countries. Right: WPAI per MRC-score; Absenteeism *N* = 260, presenteeism *N* = 268, overall work impairment *N* = 259, activity impairment (270), total activity impairment *N* = 1039

For data-orientation, univariable regressions are presented in Additional file 1: Appendix 4 Table E3. In each of the individual country multivariable analyses and in the meta-analysis, breathlessness severity (MRC-score) was identified as a robust predictor for activity impairment (Fig. 3, 4, 5, Additional file 1: Appendix 4 Table E4). Other significant predictors in the meta-analyses were working (inversely related), smoking, and solid fuel use. The results were similar for both meta-analyses (i.e. regardless of excluding Kyrgyzstan from the analysis). Besides activity impairment, MRC-score was identified as a predictor for both presenteeism and overall work impairment. In contrast, absenteeism remained relatively low, independent of MRC-score (Fig. 5b).

## Discussion

We have evaluated the socioeconomic burden of CLD in low-resource settings across the globe, with a specific focus on work- and activity impairment and its risk factors. Our findings demonstrate substantial disease-related productivity impairment, overall work impairment, and activity impairment. Remarkably, absenteeism consistently remained relatively low. Severity of breathlessness, smoking, and solid fuel use were modifiable predictors for impairment.

The patterns of absenteeism and presenteeism we have identified are similar in high-resource settings [23]. However, in our study, absolute WPAI-scores were considerably higher for all outcomes but absenteeism. Possibly, absenteeism remains low in low-resource settings, because limited or non-existent social security systems [2, 18] ‘force’ employees to show up at work, at the expense of a decreased productivity. Note that all WPAI-data should be interpreted within their context. The different sample sizes and diverse population characteristics would not allow for direct comparison of WPAI-scores between countries. Although participants were included using similar methods, countries and settings were selected based on diversity. The diversity-based selection resulted, for example, in differences in proportions of COPD-patients and breathlessness severity. To illustrate, Kyrgyz WPAI-scores were high compared to scores in the other countries; breathlessness severity (a strong predictor) was high in Kyrgyzstan too. Breathlessness severity could be high because of lower ambient oxygen levels in the mountains. Absenteeism could particularly be impacted by the extreme temperatures (− 20 °C in winter) and rough Kyrgyz terrains in the Kyrgyz setting, forming barriers to travelling to/from work. Hence, only considered within this context, Kyrgyz WPAI-outcomes provide meaningful information, based on real-world data on CLD-related impairment [39].

Severity of breathlessness was already reported as a predictor for impairment for higher-resource settings [36, 40, 41]; we are the first to confirm this for low-resource settings. Besides activity impairment, also presenteeism and overall work impairment increased linearly with MRC-scores. Meanwhile, absenteeism consistently remained remarkably low despite severe breathlessness. This seems plausible, as most people with severe breathlessness have stopped working (severe breathlessness was significantly more common in our non-working population). Yet, if employed, again they ensure not to miss worktime as social security is limited.

We argue severe breathlessness may be inherent to low-resource settings, because access to healthcare and adequate equipment is limited in low-resource settings [2, 3, 16]. Therefore, a) possibly only the more severely ill patients receive a spirometry-confirmed CLD-diagnosis (one of our inclusion criteria) and b) undertreatment is common and could trigger severe symptoms. Second, low-resource settings have higher and earlier exposure to behavioral and environmental risk factors [3, 5–7, 10, 14, 42] (45% of our Kyrgyz population smoked, and the entire rural population relies on solid fuels for cooking and heating) [8]. This can result in more severe disease and hence, more breathlessness. Interestingly, higher and earlier exposure to risk factors in lower-resource settings also lead to earlier onset of disease. In combination with lower life-expectancies in lower-resource countries, this explains why the age of the patient population in Uganda was generally lower than in Vietnam and Kyrgyzstan, where in turn it was lower than in Greece.

In addition to breathlessness, both tobacco- and solid fuel use were identified as modifiable risk factors for impairment. Tobacco use was already known to predict CLD-related impairment in high-resource settings, whereas solid fuel use is newly identified and typical for low-resource settings. Furthermore, ‘working’ was a protective factor for activity impairment. Similarly, higher activity impairment for part-time compared to fulltime employees was reported in a high-resource setting [35]. Of note, age was a significant predictor in univariable regression analyses but turned insignificant in the multivariable analyses. We assume the effect of ‘age’ diminished in the multivariable model because of the presence of the more accurate predictor ‘breathlessness severity’ (and commonly, like age, breathlessness severity increases over time).

Comparison of CLD-related impairment to impairment due to other chronic diseases in low-resource settings is difficult due to a paucity of data. A large systematic review reported on more than 80 studies assessing WPAI due to chronic disease, yet the settings described were almost exclusively in high-income countries. The handful of studies that also included low-income countries did not report their results separately for the low-income countries [23].

To our knowledge, this is the first large (*N* > 1000) study to focus on the socioeconomic burden of CLDs in low-resource settings across the world providing data from validated and well-accepted instruments (WPAI, MRC-scale). This paper furthermore answers the call for robust studies identifying modifiable predictors for CLD-related impairment [25]. While some predictors were previously reported for high-resource settings [35–37, 40], we have identified a predictor specifically relevant to low-resource settings: solid fuel use. Another strength of our study is the use of identical, yet contextually tailored, methods across four diverse settings, (Additional file 1: Appendix 2 Table E1) improving the fit with the local situation. Some limitations should also be noted. The inclusion of only spirometry-confirmed CLD-patients might lead to selection bias; in low-resource settings patients possibly seek costly healthcare when more severely-ill, and when more severely-ill, impairment scores are higher [17, 43]. Yet given frequent CLD-misdiagnosis in the absence of spirometry [44], particularly in low-resource settings, we valued this criterion. On the one hand, misclassification due to variable spirometry interpretation or other causes for airflow obstruction (post-tuberculosis, childhood respiratory infections) cannot be fully ruled out. On the other hand, other causes would result in the need for similar interventions: reduction of occupational and household air pollution, smoking cessation, pulmonary rehabilitation, etc. Also, we had no control group in our study while socioeconomic data in low-resource settings are scarce; this made it difficult to compare our results to a healthy population. Besides, the actual population-based socioeconomic impact may be underestimated in our study. People frequently missing work might be forced to leave, particularly in more physically demanding jobs as is common in low-resource settings. Unfortunately, we cannot derive the number of early-retirements due to CLD from our data. Lastly, following the WPAI-questionnaire in its validated form, we only assessed absenteeism and presenteeism for those working for an income. We recommend future studies to apply all relevant questions not only to those “currently employed (working for pay)”, but also to those self-employed or working for subsistence, as is common in LMICs.

Nevertheless, the substantial WPAI-scores we have observed imply a high socioeconomic burden due to CLDs in low-resource settings. Considering widescale underdiagnosis of CLD, particularly in low-resource settings, costs may be even higher than policymakers may realize [45, 46]. The risk factors we identified could provide potential leads for combatting impairment. Policymakers could introduce awareness-programs to educate populations on the risks of tobacco- and solid fuel use, and on affordable solutions (e.g. clean stoves). Furthermore, enhanced self-management and pulmonary rehabilitation programs could benefit the factor breathlessness severity [47]. Self-management could be challenging in low-resource settings due to more scarce availability of medications, limited access to healthcare, or widespread overestimations on disease control [3, 4, 48, 49]. Medications should therefore be available at economic costs [50], health infrastructures need to facilitate continuity of care [4], and healthcare workers should educate patients on disease control. Concurrently, although four diverse low-resource settings were assessed in our study, causality and generalizability of our findings should be evaluated further.

## Conclusions

Our results showed that although relatively limited worktime was missed due to CLD in low-resource settings, the disease related productivity- and activity impairment was substantial. Severity of breathlessness, smoking, and solid fuel use were significant modifiable risk factors for impairment. Our results warrant increased awareness on the impact of CLD and the risk factors, preventive actions regarding tobacco and solid fuel use, and enhanced clinical management of CLD in low-resource settings by healthcare workers, policymakers, patients, and employers alike.

## Supplementary information

**Additional file 1:** STROBE checklist, Methods in detail, Questionnaire, Outcomes detailed per country.

## Data Availability

Individual de-identified participant data and meta-data can be shared upon reasonable request. This includes the study protocol, data-dictionaries with details on data cleaning and meeting minutes describing considerations for data analysis. Within reasonable time after email request data will be shared via a secure webbased system.

## Abbreviations

ACO: Asthma-COPD overlap
CLD: Chronic lung disease
COPD: Chronic obstructive pulmonary disease
FRESH AIR: Free Respiratory Evaluation and Smoke-exposure reduction by primary Health cAre Integrated gRoups
IQR: Interquartile range
MRC: Medical research council
WPAI: Work productivity and activity impairment
