# Supplementary material for: The socioeconomic burden of chronic lung disease in low-resource settings across the globe – an observational FRESH AIR study
Source: Respir Res. 2019 Dec 21;20:291. doi: 10.1186/s12931-019-1255-z (PMC6925865; doi:10.1186/s12931-019-1255-z)
Supplement: Supplementary file 1 — Additional file 1: STROBE checklist, Methods in detail, Questionnaire, Outcomes detailed per country. [file 12931_2019_1255_MOESM1_ESM.pdf]

Online Supplement

The socioeconomic burden of chronic lung disease in low-resource settings across the globe – an observational FRESH AIR study

Evelyn A. Brakema, MD; Aizhamal Tabyshova, MD; Rianne MJJ van der Kleij, Ass Prof; Talant Sooronbaev, Prof, MD; Christos Lionis, Prof, MD; Marilena Anastasaki, Msc; An Pham Le, MD; Luan Than Nguyen, MD; Bruce Kirenga, MD; Simon Walusimbi, MD, PhD; Maarten J Postma, Prof; Niels H Chavannes, Prof, MD; Job FM van Boven, Ass Prof, On behalf of the FRESH AIR collaborators

Appendix 1: STROBE checklist (P2)

Appendix 2: The methods section in more detail (P6)

Appendix 3: Questionnaire (P14)

Appendix 4: Outcomes detailed per country (P21)

APPENDIX 1 STROBE CHECKLIST

STROBE Statement—Checklist of items that should be included in reports of ***cross-sectional studies***

	Item No	Recommendation	Page	If applicable, relevant section or text from manuscript
**Title and abstract**	1	(*a*) Indicate the study's design with a commonly used term in the title or the abstract	1,3	"The socioeconomic burden of chronic lung disease in low-resource settings across the globe – an observational FRESH AIR study"<br>"We performed a cross-sectional, observational FRESH AIR study in Uganda, Vietnam, Kyrgyzstan, and Greece."
		(*b*) Provide in the abstract an informative and balanced summary of what was done and what was found	3	See Abstract
**Introduction**				
Background/rationale	2	Explain the scientific background and rationale for the investigation being reported	5,6	See Introduction
Objectives	3	State specific objectives, including any prespecified hypotheses	3,6	"Therefore, we aimed to estimate the chronic lung disease -related socioeconomic burden in diverse low-resource settings across the globe." (p3)<br><br>"Therefore, the aim of this study was to estimate the chronic lung disease -related socio-economic burden in low-resource settings across the globe. To inform governmental and public health policy, we focused on work productivity and activity impairment and its modifiable clinical and environmental risk factors." (p6)
**Methods**				
Study design	4	Present key elements of study design early in the paper	6,7(-9)	See Methods, particularly Design and setting
Setting	5	Describe the setting, locations, and relevant dates, including periods of recruitment, exposure, follow-up, and data collection	6,7(-9)<br>Appendix 2	See Methods: particularly Design and setting

Participants	6	(*a*) Give the eligibility criteria, and the sources and methods of selection of participants	7, Appendix 2	See Methods: Participants
Variables	7	Clearly define all outcomes, exposures, predictors, potential confounders, and effect modifiers. Give diagnostic criteria, if applicable	7-9, Appendix 2	See Methods: Instruments, Statistical Analysis
Data sources/ measurement	8*	For each variable of interest, give sources of data and details of methods of assessment (measurement). Describe comparability of assessment methods if there is more than one group	7,8, Appendix 2	See Methods: Procedures, Instruments
Bias	9	Describe any efforts to address potential sources of bias	6-9	See Methods: amongst others but not limited to standardized procedures, validated questionnaires, adjusted regression analyses.
Study size	10	Explain how the study size was arrived at	8	See Methods: Sample size
Quantitative variables	11	Explain how quantitative variables were handled in the analyses. If applicable, describe which groupings were chosen and why	8,9, Appendix 2	See Methods: Statistical analysis
Statistical methods	12	(*a*) Describe all statistical methods, including those used to control for confounding	8,9, Appendix 2	See Methods: Statistical analysis
		(*b*) Describe any methods used to examine subgroups and interactions	8,9, Appendix 2	See Methods: Statistical analysis
		(*c*) Explain how missing data were addressed	8,9, Appendix 2	See Methods: Statistical analysis
		(*d*) If applicable, describe analytical methods taking account of sampling strategy	8,9, Appendix 2	See Methods: Statistical analysis
		(*e*) Describe any sensitivity analyses	Not applicable	Not applicable

**Results**				
Participants	13*	(a) Report numbers of individuals at each stage of study—eg numbers potentially eligible, examined for eligibility, confirmed eligible, included in the study, completing follow-up, and analyzed	10	See Figure 1
		(b) Give reasons for non-participation at each stage	10	See Figure 1
		(c) Consider use of a flow diagram	10	See Figure 1
Descriptive data	14*	(a) Give characteristics of study participants (eg demographic, clinical, social) and information on exposures and potential confounders	9,10	See Results: Clinical and demographic characteristics, Table 1, and Appendix 4 Table E1
		(b) Indicate number of participants with missing data for each variable of interest	10,11 Appendix 4	See Figure 1-5, Table 1. Appendix 4
Outcome data	15*	Report numbers of outcome events or summary measures	11, Appendix 4	See Results: Work productivity and activity impairment onwards, Figure 2-5, Appendix 4
Main results	16	(*a*) Give unadjusted estimates and, if applicable, confounder-adjusted estimates and their precision (eg, 95% confidence interval). Make clear which confounders were adjusted for and why they were included	11, Appendix 4	See Results: Work productivity and activity impairment onwards, Figure 2-5. Appendix 4
		(*b*) Report category boundaries when continuous variables were categorized	Not applicable	Not applicable
		(*c*) If relevant, consider translating estimates of relative risk into absolute risk for a meaningful time period	Not applicable	Not applicable
Other analyses	17	Report other analyses done—eg analyses of subgroups and interactions, and sensitivity analyses	11 Appendix 4	See Results: subgroup analyses done for:<br>Impairment per country ('Predictors for activity impairment' and Figure 5A, Appendix 4)<br>Impairment per MRC score ('Predictors for activity impairment' and Figure 5B)

**Discussion**				
Key results	18	Summarize key results with reference to study objectives	12	See Discussion: first paragraph
Limitations	19	Discuss limitations of the study, taking into account sources of potential bias or imprecision. Discuss both direction and magnitude of any potential bias	14	See Discussion: main paragraph p14
Interpretation	20	Give a cautious overall interpretation of results considering objectives, limitations, multiplicity of analyses, results from similar studies, and other relevant evidence	(12-15), 15	See Discussion: particularly last paragraph
Generalizability	21	Discuss the generalizability (external validity) of the study results	15	See Discussion: “Concurrently, although four diverse low-resource settings were assessed in our study, causality and generalizability of our findings should be evaluated further.”
**Other information**				
Funding	22	Give the source of funding and the role of the funders for the present study and, if applicable, for the original study on which the present article is based	16	“This study was funded by the EU Research and Innovation program Horizon2020 (Health, Medical research and the challenge of ageing) under grant agreement no. 680997. The funders had no role in study design, data collection, data analysis, data interpretation, or writing of the report. All authors had full access to all the data and EB, and JB had the final responsibility for the decision to submit the study for publication.”

*Give information separately for exposed and unexposed groups.

Note: An Explanation and Elaboration article discusses each checklist item and gives methodological background and published examples of transparent reporting. The STROBE checklist is best used in conjunction with this article (freely available on the Web sites of PLoS Medicine at http://www.plosmedicine.org/, Annals of Internal Medicine at http://www.annals.org/, and Epidemiology at http://www.epidem.com/). Information on the STROBE Initiative is available at www.strobe-statement.org.

APPENDIX 2 THE METHODS SECTION IN MORE DETAIL

METHODS

This study was part of the FRESH AIR (Free Respiratory Evaluation and Smoke-exposure reduction by primary Health cAre Integrated gRoups) project (trial registration number: NTR5759), that aimed to address the prevention, diagnosis, treatment, and its implementation regarding CLDs in low-resource settings [E1]. This study is reported according to STROBE guidelines (Appendix 1).

Design and setting

This observational, cross-sectional study was performed between July 2016 and March 2018 in Uganda, Vietnam, Kyrgyzstan, and rural Greece.

Setting were considered to be ‘low-resource’ when a lack of resources (often financial means) at societal level led to disadvantaged circumstances such as limited infrastructure, limited access to equipment and medication, and/or limited trained personnel. The study sites were sampled purposefully to represent four distinct low-resource settings in terms of geography, ethnicity, risk factor exposure, and healthcare- and political system. At these sites, we purposefully selected healthcare centers that routinely used spirometry for the diagnosis of CLDs (asthma, COPD, or asthma-COPD overlap (ACO)). The exact selection method of settings and participants was designed in close collaboration with the local teams to meet their daily clinical routine, typical patient population, and available resources (Table E1). For example, although we generally aimed for a proportion of out- and inpatients representative to each national CLD population, we only recruited outpatients in Greece. We recruited from rural primary care facilities other than urban hospitals in

Greece, to maintain focus on low-resource settings, and rural Greek facilities serve outpatients only [E2]. Meanwhile, in Uganda, Vietnam, and Kyrgyzstan we recruited from hospitals, as the primary care facilities have no spirometry.

All but one of the invited centers were willing to participate; one hospital in Vietnam declined due to a concomitant time-consuming hospital quality assessment. A similar district health center consented to participate instead.

Participants

We recruited participants consecutively during visits to the selected health centers. In three Kyrgyz hospitals, the existence of complete and up-to-date patient registries enabled random sampling via a registry instead (Table E1). We included patients aged 15 years or older with a spirometry-confirmed diagnosis of COPD, asthma, or ACO. Each setting used the COPD definition as recommended by the Global Initiative for Chronic Obstructive Lung Disease (GOLD): a post-bronchodilator FEV_1/FVC ratio of <70% [E3]. We did not deploy any additional inclusion criteria for COPD patients regarding age or tobacco use, as patients in low-resource settings may develop COPD at a younger age due to early life disadvantage factors such as household air pollution (regardless of tobacco use) [E4, 5-9]. An asthma definition was based on a physician confirmed clinical diagnosis according to the Global Initiative for Asthma (GINA) guidelines, and ACO as having both COPD and asthma [E10]. Patients with a disability hampering communication and/or proper understanding of the study, patients too severely ill to participate or with missing outcomes on activity impairment, were excluded.

Procedures

Eligible study participants were identified and informed about the study by their regular physicians during a routine visit (Table E1). Participants then provided consent to a research

Table E1 Study settings and their tailored recruitment/interview procedure.

Country	Setting	Explanation	Specifications in the recruitment- and interview procedure
Uganda	Kampala; a referral hospital, annex of a national referral hospital.[1] Out- and inpatients.	Until 2015, this was the only national site where asthma and COPD were diagnosed using spirometry with qualified pulmonologists and research staff.	A spirometry technician and a clinician who worked at the site took the role of research assistant.
Vietnam	South Vietnam; Pulmonology department of one academic and four district hospitals.[2] Out- and inpatients.	The combination of an academic hospital with district hospitals is representative for the rural and urban Ho Chi Minh City area.	Either nurses or physicians from the local pulmonology department took the role of research assistant. Outpatients were recruited after their consultation, while they were in preparation to receive their medication at the Hospital Drug Store. Inpatients were invited to participate while in preparation for their discharge. Hence, if eligible participants were transferred to another hospital and not discharged to their home, they were not included.
Kyrgyzstan	Pulmonology departments of the five major hospitals in Kyrgyzstan.[3] Out- and inpatients.	Five sites across the country to have an optimal representation of patients from both urban and rural provinces.	The patient registries in the three hospitals in Bishkek allowed for more efficient sampling: instead of consecutive recruitment during patient visits, every third patient from the hospitals' pulmonology registries was invited. Research assistants invited participants and administered the questionnaire via telephone. Consent was provided verbally.

Greece	Rural Crete (Heraklion); two primary health care centers and eight satellite practices.[4] Only outpatients.	Due to the focus on low-resource settings, only rural sites were included which have no inpatient facilities.	Family physicians took the role of research assistants. In some practices, spirometry was not available. A CLD diagnosis was then assured by a spirometry-confirmed diagnosis elsewhere as recorded in the medical records.

[1] Kiruddu General Referral Hospital, an annex of the Mulago National Referral hospital.
[2] University Medical Center in Ho Chi Minh (academic hospital); District 2 hospital, District 4 hospital, Tan Phu District hospital, and Hoc Mon District Hospital.
[3] Bishkek – National Centre of Cardiology and Internal Medicine; National Hospital; Town Clinical Hospital number 6; Osh – Osh Joint Regional Hospital; Jalal Abad – Jalal Abad Regional Hospital.
[4] Exact names of centers not provided to ensure anonymity of the patients in these smaller rural practices.

assistant, and subsequently filled out a questionnaire (detailed in the following section). Their physician added the clinical data from existing medical history files. The research assistant remained available for assistance and checked the questionnaire upon completion, to ensure misinterpretations or missing data were immediately identified and corrected by the participant or physician. In three hospitals in Kyrgyzstan, well-organized patient registries allowed the research assistants to recruit participants and to administer the questionnaire per telephone. All research assistants or other involved collaborators (e.g. physicians) received a training on the study objectives, methods and procedures. No personal information that could identify the patient was collected (such as date of birth, name or address).

Instruments

The questionnaire was composed of several validated [E11, 12], structured questionnaires with additional open-ended questions, assessing demographic, socio-economic, and health factors (Appendix 3). Symptom severity was classified by the Medical Research Council (MRC) breathlessness scale, ranging from 1-5 with higher

scores indicating more severe breathlessness [E11]. The outcome work- and other activity impairment was assessed using the recommended Work Productivity- and Activity Impairment (WPAI) questionnaire [E12, 13, 14]. The WPAI-questionnaire assesses CLD -related absenteeism (work time missed), presenteeism (decreased productivity while working), overall work impairment (absenteeism and presenteeism combined), and impairment of regular activities during the preceding seven days [E12]. All items are calculated into percentages (Appendix 3), resulting in a scale from 0-100%, with higher numbers indicating worse outcomes (greater impairment and less productivity). When available, we used official, validated WPAI-translations.[E15]

All questions were provided to participants in the local language (English, Vietnamese, Russian, Greek). In Uganda, where several local languages are spoken, the involved medical workers and research assistants represented all major language groups. We piloted the full questionnaire in small samples and improved the initial translation and contextual adaptations accordingly. For example, we noticed many patients were not aware of the name of their lung disease, so we added clarifications on this before asking about the impact of their ‘COPD’ and/or ‘asthma’.

Sample size

Notably, data collection in settings without proper electronic patient files, limited equipment, and often lower efficiency can be relatively challenging compared to high-resource settings. Therefore, taking the local circumstances and our budget into account, we carefully considered the targeted sample size to be both feasible and informative. With a total covered population of +/-146 million (Uganda: 40; Kyrgyzstan: 6; Vietnam: 90; Greece:10 million), an estimated global CLD prevalence

of 5% (based on previous studies [E16, 17]), a number of 1040 participants resulted in a 99% confidence level and a 4% error margin. Notably, this study was performed in four different low-resource settings with mostly unknown CLD prevalence. Therefore, the sample size was not calculated with the intention to compare the four countries and was not weighted based on country size or differences in CLD prevalence.

Statistical analysis

Data were analyzed using SPSS version 25 (IBM, Armonk, NY, USA). Descriptive statistics (proportions, confidence intervals, medians and interquartile ranges) were used to examine population characteristics and the WPAI. For data orientation only, we first assessed the relation between predictors and activity impairment per country using univariable linear regression. We then performed a forced entry multivariable linear regression per country (excluding missing variables pairwise). In order to meta-analyze the data, we used an identical model in each country, including sex, working status, MRC-scale, presence of at least one comorbidity, having children (instead of marital status), education level, and smoking status, while adjusting for age [E18, 19, 20]. The variables we were not able to test were income (we were hindered by missing data and registration errors), exacerbations (Uganda had no cases of reported exacerbations), and ethnicity (due to our totally different settings). We added the use of solid fuel for cooking/heating to our model, as next to smoking this is a major risk factor for CLD in low-resource settings [E8, 21, 22, 23]. There were no indications for multicollinearity. The unstandardized coefficients of each country with their 95% confidence intervals (CI) were then meta-analyzed. Because our Kyrgyz population had no asthma patients, we did a separate meta-analysis without this

country to check for any differences (Appendix 4 Table E5). We used a fixed-model for most variables in Comprehensive Meta-Analysis version 3 (Biostat, Englewood, NJ, USA). As there were indications for heterogeneity of effect between the countries for the variable ‘comorbidity’, we used a random-effect model for this variable. Coefficients with 95%CI excluding 1 were considered statistically significant.

REFERENCES

1. Cragg L, Williams S, Chavannes NH. FRESH AIR: an implementation research project funded through Horizon 2020 exploring the prevention, diagnosis and treatment of chronic respiratory diseases in low-resource settings. *NPJ Prim Care Respir Med* 2016: 26: 16035.
2. Eurostat. Statistics on rural areas in the EU. 17 Nov 2017 [cited 15 Dec 2017]; Available from: http://ec.europa.eu/eurostat/statistics-explained/index.php/Statistics_on_rural_areas_in_the_EU
3. Fabbri LM, Hurd SS, Committee GS. Global Strategy for the Diagnosis, Management and Prevention of COPD: 2003 update. *Eur Respir J* 2003: 22(1): 1-2.
4. van Gemert F, Kirenga B, Chavannes N, Kamya M, Luzige S, Musinguzi P, Turyagaruka J, Jones R, Tsiligianni I, Williams S, de Jong C, van der Molen T. Prevalence of chronic obstructive pulmonary disease and associated risk factors in Uganda (FRESH AIR Uganda): a prospective cross-sectional observational study. *Lancet Glob Health* 2015: 3(1): e44-51.
5. Lopez-Campos JL, Tan W, Soriano JB. Global burden of COPD. *Respirology* 2016: 21(1): 14-23.
6. Martinez FD. Early-Life Origins of Chronic Obstructive Pulmonary Disease. *N Engl J Med* 2016: 375(9): 871-878.
7. Lee AG, Kaali S, Quinn A, Delimini R, Burkart K, Opoku-Mensah J, Wylie BJ, Yawson AK, Kinney PL, Ae-Ngibise KA, Chillrud S, Jack D, Asante KP. Prenatal Household Air Pollution is Associated with Impaired Infant Lung Function with Sex-Specific Effects: Evidence from GRAPHS, a Cluster Randomized Cookstove Intervention Trial. *Am J Respir Crit Care Med* 2018.
8. Sood A, Assad NA, Barnes PJ, Churg A, Gordon SB, Harrod KS, Irshad H, Kurmi OP, Martin WJ, 2nd, Meek P, Mortimer K, Noonan CW, Perez-Padilla R, Smith KR, Tesfaigzi Y, Ward T, Balmes J. ERS/ATS workshop report on respiratory health effects of household air pollution. *Eur Respir J* 2018: 51(1).
9. Milanzi EB, Koppelman GH, Smit HA, Wijga AH, Oldenwening M, Vonk JM, Brunekreef B, Gehring U. Air pollution exposure and lung function until age 16 years: the PIAMA birth cohort study. *Eur Respir J* 2018: 52(3).
10. Global Initiative for Asthma. Global Strategy for Asthma Management and Prevention. 2016.
11. Bestall JC, Paul EA, Garrod R, Garnham R, Jones PW, Wedzicha JA. Usefulness of the Medical Research Council (MRC) dyspnoea scale as a measure of disability in patients with chronic obstructive pulmonary disease. *Thorax* 1999: 54(7): 581-586.
12. Reilly MC, Zbrozek AS, Dukes EM. The validity and reproducibility of a work productivity and activity impairment instrument. *Pharmacoeconomics* 1993: 4(5): 353-365.
13. Akinbami LJ, Sullivan SD, Campbell JD, Grundmeier RW, Hartert TV, Lee TA, Smith RA. Asthma outcomes: healthcare utilization and costs. *J Allergy Clin Immunol* 2012: 129(3 Suppl): S49-64.
14. Prasad M, Wahlqvist P, Shikiar R, Shih YC. A review of self-report instruments measuring health-related work productivity: a patient-reported outcomes perspective. *Pharmacoeconomics* 2004: 22(4): 225-244.
15. Reilly Associates. WPAI Translations. 2015 [cited 12 December 2015; Available from: http://www.reillyassociates.net/WPAI_Translations.html

16. Fletcher MJ, Upton J, Taylor-Fishwick J, Buist SA, Jenkins C, Hutton J, Barnes N, Van Der Molen T, Walsh JW, Jones P, Walker S. COPD uncovered: an international survey on the impact of chronic obstructive pulmonary disease [COPD] on a working age population. *BMC Public Health* 2011: 11: 612.
17. Rennard S, Decramer M, Calverley PM, Pride NB, Soriano JB, Vermeire PA, Vestbo J. Impact of COPD in North America and Europe in 2000: subjects' perspective of Confronting COPD International Survey. *Eur Respir J* 2002: 20(4): 799-805.
18. DiBonaventura M, Paulose-Ram R, Su J, McDonald M, Zou KH, Wagner JS, Shah H. The impact of COPD on quality of life, productivity loss, and resource use among the elderly United States workforce. *COPD* 2012: 9(1): 46-57.
19. Gruenberger JB, Vietri J, Keininger DL, Mahler DA. Greater dyspnea is associated with lower health-related quality of life among European patients with COPD. *Int J Chron Obstruct Pulmon Dis* 2017: 12: 937-944.
20. Pavord ID, Mathieson N, Scowcroft A, Pedersini R, Isherwood G, Price D. The impact of poor asthma control among asthma patients treated with inhaled corticosteroids plus long-acting beta2-agonists in the United Kingdom: a cross-sectional analysis. *NPJ Prim Care Respir Med* 2017: 27(1): 17.
21. World Health Organization. Chronic respiratory diseases. 2018 [cited 8 Jan 2018; Available from: http://www.who.int/respiratory/en/
22. Brakema EA, Tabyshova A, Kasteleyn MJ, Molendijk E, van der Kleij R, van Boven JFM, Emilov B, Akmatalieva M, Mademilov M, Numans ME, Williams S, Sooronbaev T, Chavannes NH. High COPD prevalence at high altitude: does household air pollution play a role? *Eur Respir J* 2019: 53(2).
23. Zhao J, Li M, Wang Z, Chen J, Zhao J, Xu Y, Wei X, Wang J, Xie J. Role of PM2.5 in the development and progression of COPD and its mechanisms. *Respir Res* 2019: 20(1): 120.
24. Heffler E, Crimi C, Mancuso S, Campisi R, Puggioni F, Brussino L, Crimi N. Misdiagnosis of asthma and COPD and underuse of spirometry in primary care unselected patients. *Respir Med* 2018: 142: 48-52.

APPENDIX 3 QUESTIONNAIRE

PERSONAL INFORMATION (TO BE COMPLETED BY PATIENTS)

Patient ID:		
Has patient previously participated in the study? *No* Yes		
DEMOGRAPHIC INFORMATION		
D1	*Age*	……… *(years)*
D2	*Gender*	*Male* *Female*
D3	*Length*	……… cm
D4	*Weight*	……… kg
D5	*Hometown*	*City (name city:……..)*<br>*Rural (name area*:………..)
D6	*Highest education*	*None*<br>*Primary*<br>*Secondary*<br>*Tertiary*<br>*Undergradutate*<br>*Postgraduate*
D7	*Occupation*	*Unemployed*<br>*Retired*<br>*Housewife,Housekeeper*<br>*Student*<br>*Civil servant*<br>*Private sector*<br>*Bussiness*<br>*Worker*<br>*Farmer*<br>*Others, namely..................................*
D8	*Income per month*	……… UGX
D9	*Children*	Yes/no
D10	*If yes, do you have to take care of them currently?*	*Yes* *No*
D11	*Do you have (private) Health Insurance?*	*Yes* *No*
D12	*Disability status*	*Yes* *No*
D13	*How long do you have to travel to the hospital?*	……… hours
RISK FACTORS		
R1	*To be exposed to smoke or dust at work area*	*Yes* *No*
R2	*To be exposed to smoke or dust at home area*	*Yes* *No*
R3	*To have family members with COPD*	*Yes* *No*
R4	*To have family members with lung cancer*	*Yes* *No*
R5	*To have family members with TB*	*Yes* *No*
R6	*To have family members smoking*	*Yes* *No*

R7	*Did you ever smoke cigarettes?*	☐ *Yes* ☐ *No (➔ R10)*
R8	*If yes, at what age did you start?*	………
R9	*How many cigarettes per day?*	………
R9a	*Did you ever smoke during pregnancy?*	☐ *Yes* ☐ *No*
R10	*Do you use electronic cigarettes?*	☐ *Yes* ☐ *No (➔ R15)*
R11	*How often?*	………
R12	*Do you use a(water/tobacco) pipe?*	☐ *Yes* ☐ *No*
R13	*How often?*	………
R14	*In case you quit, at what age did you quit?*	………
R15	*Using biomass for cooking?*	☐ *Yes* ☐ *No (➔ R16)*
R151	*If yes, kind of biomass*	☐ *Charcoal*<br>☐ *Wood*<br>☐ *Dung*<br>☐ *Others, namely*......................................
R152	*How often?*	☐ *1-2 days per week*<br>☐ *3-4 days per week*<br>☐ *Every day*
R153	*How many hours per day?*	………
R16	*Using biomass for heating in cold weather?*	☐ *Yes* ☐ *No (➔ C1)*
R161	*If yes, kind of biomass*	☐ *Charcoal*<br>☐ *Wood*<br>☐ *Dung*<br>☐ *Others, namely*....................................
R162	*How often?*	☐ *1-2 days per week*<br>☐ *3-4 days per week*<br>☐ *Every day*
R163	*How many hours per day?*	………
CLINICAL VARIABLES		
C1	*Did your doctor ever tell you that you have Asthma?*	☐ *Yes* ☐ *No*
C1.1	*Did your doctor ever tell you that you have COPD?*	☐ *Yes* ☐ *No*
C2	*What is your lung function in terms of forced expiratory volume in 1 second (in liters)*<br>*(ask doctor)*	.......liters
C2.1	*What is your lung function in terms of forced expiratory volume in 1 second (% predicted)*<br>*(ask doctor)*	……% predicted
C3	*Last week, were you experiencing any of these respiratory symptoms:*<br>*Chronic cough*<br>*Sputum production*	 <br>☐ *Yes* ☐ *No*<br>☐ *Yes* ☐ *No*

	Fatigue<br>*Breathlessness*<br>*Chest tightness*<br>*Wheezing*	*Yes* *No*<br>*Yes* *No*<br>*Yes* *No*<br>*Yes* *No*
C4	*Do you have other diseases?*	*Yes* *No (➔C5)*
C41	*If yes, which ones? (if unsure, ask doctor)*	*Hypertension*<br>*Diabetes*<br>*TB*<br>*Heart diseases*<br>*Others* (*Please specify disease)*<br>Other1:<br>Other2:<br>Other3:
C5	*How many times did you visit a medical (family) doctor for your asthma/COPD in the last 3 months?*	……… *(If 0, ➔ C6)*
C51	*Was it fully paid by your health insurance?*	*Yes(➔C6)* *No*
C52	*If not, how much did you have to pay?*	……… UGX
C6	*How many times did you visit a pulmonologist for your asthma/ COPD in the last 3 months?*	……… *(If 0, ➔ C7)*
C61	*Was it fully paid by your health insurance?*	*Yes(➔C7)* *No*
C62	*If not, how much did you have to pay?*	……… UGX
C7	*Last 3 months, how many times did the ambulance or family doctor were at your home because of asthma/COPD emergency (exacerbation)?*	……… *(If 0, ➔ C8)*
C71	*Was it fully paid by your health insurance?*	*Yes(➔C8)* *No*
C72	*If not, how much did you have to pay?*	……… UGX
C8	*How many days were you provided Intensive Care for asthma/COPD in the last year?*	……… *(If 0, ➔ C9)*
C81	*Was it fully paid by your health insurance?*	*Yes(➔C9)* *No*
C82	*If not, how much did you have to pay?*	……… UGX
C9	*How many days were you in the hospital for asthma/COPD in the last year?*	……… *(If 0, ➔ C10)*
C91	*Was it fully paid by your health insurance?*	*Yes* *No*

C92	*If not, how much did you have to pay?*	……… UGX
C10	*Did you visit a private clinic for asthma/COPD last 3 months?*	*Yes* *No(➔C11)*
C101	*If yes, how much did you have to pay?*	……… UGX
C11	*Last year, how many times were you in hospital for other reasons?*	*Yes, for….* *No*
C12	*Last year, were some of the diagnostics performed and if yes, how many times?*<br>*Spirometry*<br>*Bronchoscopy*<br>*CT-scan*<br>*X-ray*<br>*ECG*<br>Echo<br>*Ultrasound*	*Yes (….times)* *No*<br>*Yes (….times)* *No*<br>*Yes (….times)* *No*<br>*Yes (….times)* *No*<br>*Yes (….times)* *No*<br>*Yes (….times)* *No*<br>*Yes (….times)* *No*
C121	*Was it fully paid by your health insurance?*	*Yes (➔C13)* *No*
C122	*If not, how much did you have to pay?*	……… UGX
C13	*Last year, did you use any respiratory medication?*	*Yes* *No(➔C14)*
C130	*If yes, what medication for lung disease did you use (generic names)? (if unsure, ask doctor)*	1Name:……………..<br>2Name:……………...<br>3Name:……………...<br>4Name:……………...
C131	*Were they provided for free in the hospital or was it fully paid by your health insurance?*	*Yes(➔C14)* *No*
C132	*If not, how much did you have to pay?*	……… UGX
C14	*Last year, did you receive a vaccination for*<br>Influenza<br>Pneumococcal	*Yes* *No*<br>*Yes* *No*

MRC QUESTIONNAIRE

Please tick (X) into an appropriate answer with your current breath

Description	Degree
I only get breathless with strenuous exercise.	☐ 1
I get short of breath when hurrying on level ground or walking up a slight hill.	☐ 2
On level ground, I walk slower than people of the same age because of breathlessness or have to stop for breath when walking at my own pace.	☐ 3
I stop for breath after walking about 100 yards or after a few minutes on level ground.	☐ 4
I am too breathless to leave the house or I am breathless when dressing.	☐ 5

Work Productivity and Activity Impairment Questionnaire for asthma/COPD

The following questions ask about the effect of asthma/COPD on your ability to work and perform regular activities. *Please fill in the blanks or circle a number, as indicated.*

1. Are you currently employed (working for pay)? _____ NO ___ YES
   If NO, check "NO" and skip to question 6.

The next questions are about the **past seven days**, not including today.

2. During the past seven days, how many hours did you miss from work because of problems associated with asthma/COPD? *Include hours you missed on sick days, times you went in late, left early, etc., because of your PROBLEM. Do not include time you missed to participate in this study.*

   _____ HOURS

3. During the past seven days, how many hours did you miss from work because of any other reason, such as vacation, holidays, time off to participate in this study?

   _____HOURS

4. During the past seven days, how many hours did you actually work?

   _____HOURS *(If "0", skip to question 6.)*

5. During the past seven days, how much did asthma/COPD affect your productivity while you were working?

Think about days you were limited in the amount or kind of work you could do, days you accomplished less than you would like, or days you could not do your work as carefully as usual. If asthma/COPD affected your work only a little, choose a low number. Choose a high number if asthma/COPD affected your work a great deal.

Consider only how much asthma/COPD affected productivity while you were working.

Asthma/COPD had no effect on my work	0	1	2	3	4	5	6	7	8	9	10	Asthma/COPD completely prevented me from working

CIRCLE A NUMBER

6. During the past seven days, how much did asthma/COPD affect your ability to do your regular daily activities, other than work at a job?

By regular activities, we mean the usual activities you do, such as work around the house, shopping, childcare, exercising, studying, etc. Think about times you were limited in the amount or kind of activities you could do and times you accomplished less than you would like. If asthma/COPD affected your activities only a little, choose a low number. Choose a high number if asthma/COPD affected your activities a great deal.

Consider only how much asthma/COPD affected your ability to do your regular daily activities, other than work at a job.

Asthma/COPD had no effect on my daily activities	0	1	2	3	4	5	6	7	8	9	10	Asthma/COPD completely prevented me from doing my daily activities

CIRCLE A NUMBER

Calculation of the WPAI scores:

"WPAI outcomes are expressed as impairment percentages, with higher numbers indicating greater impairment and less productivity, i.e., worse outcomes, as follows:

Questions:
1 = currently employed
2 = hours missed due to specified problem
3 = hours missed other reasons
4 = hours actually worked
5 = degree problem affected productivity while working
6 = degree problem affected regular activities

Scores:
Multiply scores by 100 to express in percentages.

Percent work time missed due to problem: Q2/(Q2+Q4)

Percent impairment while working due to problem: Q5/10

Percent overall work impairment due to problem:
Q2/(Q2+Q4)+[(1-(Q2/(Q2+Q4)))x(Q5/10)]

Percent activity impairment due to problem: Q6/10"

APPENDIX 4 OUTCOMES DETAILED PER COUNTRY

Table E1 – Clinical and demographic characteristics in further detail

	Uganda<br>N = 173 (16.6%)	Vietnam<br>N = 471 (45.3%)	Kyrgyzstan<br>N = 306 (29.4%)	Greece<br>N = 90 (8.7%)	Total<br>N = 1040 (100%)
**Demographic characteristics**					
Male	39 (22.5)	274 (58.2)	188 (61.4)	55 (61.1)	556 (53.3)
Age (yrs), median [IQR]	35.0 [22.5-47.0]	62.0 [52.0-72.0]	62.0 [55.0-70.0]	72.0 [63.8-79.0]	60.0 [48.0-70.0]
Proportional to life expectancy*	0.58 [0.38-0.78]	0.82 [0.68-0.95]	0.77 [0.68-0.86]	0.89 [0.79-0.98]	0.78 [0.64-0.92]
N (%) of people of working age†	161 (93.1)	192 (40.8)	163 (53.3)	17 (18.9)	533 (51.2)
BMI (kg/m^2), median [IQR]	23.8 [20.4-28.3]	21.9 [19.5-24.4]	25.8 [23.7-29.4]	28.0 [24.7-31.5]	23.9 [20.8-27.3]
Highest education					
None	8 (4.7)	31 (6.6)	0 (0.0)	18 (20)	57 (5.5)
Primary	54 (31.4)	158 (33.5)	2 (0.7)	52 (57.8)	266 (25.6)
Secondary	64 (37.2)	126 (26.8)	13 (4.2)	16 (17.8)	219 (21.1)
Above secondary	46 (26.7)	156 (33.1)	291 (95.1)	4 (4.4)	497 (47.8)
Working status					
Working	93 (53.8)	193 (41.1)	92 (30.1)	23 (25.6)	401 (38.6)
Employed (for payment)	*81 (87.1)*	*134 (69.4)*	*40 (43.5)*	*15 (65.2)*	*270 (67.3)*
Not working	41 (23.7)	153 (32.6)	34 (11.1)	13 (14.4)	241 (23.2)
Student	36 (20.0)	5 (1.1)	0 (0.0)	0 (0.0)	41 (3.9)
Retired	3 (1.7)	119 (25.3)	180 (58.8)	54 (60.0)	356 (34.2)
Having child(ren)	117 (67.6)	417 (88.5)	302 (98.7)	79 (87.8)	915 (88.0)
Ever smoker	6 (3.5)	251 (53.3)	138 (45.1)	62 (68.9)	457 (43.9)
Pack years, median [IQR]	*3.8 [2.0-19.9]*	*29.0 [15.5-44.0]*	*27.0 [14.2-40.8]*	*57.0 [26.1-74.0]*	*30.0 [15.1-45.0]*
Male	*4 (66.7)*	*234 (93.2)*	*134 (97.1)*	*47 (75.8)*	*419 (91.7)*
Current smoker	*6 (100.0)*	*92 (36.7)*	*37 (26.8)*	*40 (64.5)*	*175 (38.3)*
Solid fuel use	170 (98.8)	130 (27.6)	218 (71.5)	44 (49.4)	562 (54.0)
Occupational exposure‡	87 (93.5)	104 (53.9)	37 (40.2)	10 (43.5)	238 (59.4)
**Health characteristics**					
Diagnosed as					
COPD	11 (6.4)	190 (40.3)	305 (99.7)	67 (74.4)	573 (55.1)
Asthma	161 (93.1)	223 (47.3)	0 (0.0)	16 (17.8)	400 (38.5)
ACO	1 (0.6)	58 (12.3)	1 (0.3)	7 (7.8)	67 (6.4)
Breathlessness severity (MRC-scale), median [IQR]	2.0 [1.0-2.0]	3.0 [2.0-4.0]	4.0 [3.0-4.0]	2.0 [2.0-4.0]	3.0 [2.0-4.0]
Exacerbation(s) in past year	0 (0.0)	102 (21.7)	35 (11.4)	9 (10.0)	146 (14.0)
Comorbidity (any)	27 (15.6)	228 (48.4)	62 (20.3)	44 (48.9)	361 (34.7)
Heart disease	2 (1.2)	95 (20.2)	14 (4.6)	21 (23.3)	132 (12.7)
Diabetes	0 (0.0)	48 (10.2)	17 (5.6)	21 (23.3)	86 (8.3)
Tuberculosis	0 (0.0)	12 (2.5)	2 (0.7)	0 (0.0)	14 (1.3)
Others	25 (14.5)	119 (25.3)	33 (10.8)	16 (17.8)	193 (18.6)

*Data are in numbers (%) unless stated otherwise. BMI = body mass index; ACO = asthma-COPD overlap. MRC = medical research council. Text in italics: category within category above. *Life expectancy in years: Uganda (U) 60, Vietnam (V) 76, Kyrgyzstan (K) 71, Greece (G) 81 (Worldbank 2018). †Considered working age: U 18-60, V 15-60 (men) & 15-55 (women), K 18-63 (men) & 18-55 (women), G 18-67. ‡Regards only those working. Missing values N (%) for BMI 6 (0.6) in G; education 1 (0.1) in U; working status 1 (0.1) in V; pack years 13 (1.2) 1 in G, 2 in V, 10 in K; solid fuel use 3 (0.3) 1 in U, K, and G; MRC-score 1 (0.1) in U; exacerbation 1 (0.1) in G.*

Table E2 – Work productivity and activity impairment (WPAI) due to CLD per country

WPAI item	Uganda	N	Vietnam	N	Kyrgyzstan	N	Greece	N	Total	N
					**Employed population**					
**Absenteeism**	11.4 (6.5-16.3)	80	8.9 (5.3-12.5)	128	51.5 (37.5-65.6)	39	9.4 (1.8-17.0)	13	16.1 (12.5-19.6)	260
**Presenteeism**	21.8 (18.2-25.3)	80	19.8 (15.8-23.7)	133	55.6 (47.4-63.9)	40	16.2 (7.8-24.5)	15	25.5 (22.5-28.5)	268
**Overall work impairment**	29.2 (23.7-34.6)	79	25.4 (20.6-30.2)	128	71.3 (61.3-81.3)	39	23.2 (11.1-35.4)	13	33.3 (29.5-37.0)	259
**Activity impairment**	21.3 (17.5-25.0)	81	20.4 (16.2-24.5)	134	52.1 (44.1-60.0)	40	13.1 (3.4-22.8)	15	25.1 (22.1-28.1)	270
					**Total population**					
**Activity impairment**	25.1 (22.3-27.9)	173	34.2 (31.8-36.6)	47	56.9 (54.3-59.4)	306	44.3 (38.2-50.5)	90	40.2 (38.6-41.9)	1040

Despite the non-normal distribution, numbers are presented here as mean 95% confidence intervals, in order to facilitate interpretation of the data within the country's context. CLD = chronic lung disease. N = number of participants in the analysis. Due to different population characteristics per country, data should be interpreted within the country's context and not be used to directly compare between countries.

Table E3 – Predictors for activity impairment, univariable regression model

	Uganda N = 173		Vietnam N = 471		Kyrgyzstan N = 306		Greece N = 90	
	**B**	**95%CI**	**B**	**95%CI**	**B**	**95%CI**	**B**	**95%CI**
Male	-.61	(-7.34–6.13)	4.05	(-.78–8.88)	4.55	(-.70–9.80)	5.69	(-6.92–18.29)
Age (years)	0.17	(-.01–.36)	.47*	(.33–.61)	.31*	(.09–.54)	.94*	(.48–1.40)
BMI	.31	(-.19–.81)	-.69*	(-1.26– -.12)	.18	(-.31– .67)	2.14*	(.88–3.39)
Higher education†	-.67	(-7.07–5.72)	-11.53*	(-16.50– -6.56)	-6.80	(-18.67–5.06)	-22.85	(-52.41–6.71)
Working	-5.42	(-11.00–.17)	-13.37*	(-18.08– -8.67)	-6.24*	(-11.79– -.68)	-18.09*	(-31.71– -4.46)
Having children	4.62	(-1.36–10.59)	10.58*	(3.14–18.02)	24.69*	(2.25–47.12)	15.29	(-3.27–33.86)
Ever smoker	3.36	(-12.01–18.73)	10.75*	(6.06–15.44)	5.85*	(.73–10.96)	-9.78	(-22.95–3.39)
Solid fuel use	5.12	(-21.27–31.51)	8.89*	(3.60–14.17)	3.43	(-2.26–9.12)	10.44	(-1.74–22.63)
Diagnosed as (COPD = reference)								
Asthma	-5.28	(-16.82–6.26)	-10.35*	(-15.34– -5.36)	/	/	-18.98*	(-34.85– -3.12)
ACO	/	/	5.37	(-2.21–12.96)	/	/	-8.36	(-31.01–14.29)
Severity in MRC-scale	10.52*	(6.13–14.91)	10.26*	(8.49–12.02)	9.09*	(6.53–11.64)	14.47*	(10.28–18.66)
Exacerbation	/	/	21.82*	(16.36–27.27)	.96	(-7.11–9.03)	20.83*	(.69–40.98)
Comorbidity, at least 1	13.72*	(6.25–21.19)	3.03	(-1.75–7.81)	4.54	(-1.83–10.91)	11.98	(-.11–24.06)
Heart disease	30.26*	(4.34–56.19)	5.57	(-.36–11.51)	7.78	(-4.48–20.04)	17.95*	(3.86–32.04)
Tuberculosis	/	/	14.52	(-.59–29.63)	-6.91	(-38.76–24.95)	/	/
Diabetes	/	/	10.42*	(2.58–18.27)	1.45	(-9.76–12.66)	12.98	(-1.35–27.31)
Other	11.82*	(4.02–19.62)	-5.15	(-10.63–.33)	2.50	(-5.78–10.77)	.05	(-16.09–16.20)

*ACO = asthma-COPD overlap; BMI = body mass index; COPD = chronic obstructive pulmonary disease; MRC = medical research council breathlessness scale. *Value was considered statistically significant. †Above secondary education. / Not applicable due to limited no. of cases. Missing values N (%) for BMI 6 (0.6) in Greece; education 1 (0.1) in Uganda; working status 1 (0.1) in Vietnam; solid fuel use 3 (0.3) 1 in Uganda, Kyrgyzstan, and Greece; MRC-score 1 (0.1) in Uganda; exacerbation 1 (0.1) in Greece.*

Table E4 – Predictors for activity impairment, multivariable regression model, meta-analysis all countries

	Uganda N = 173		Vietnam N = 471		Kyrgyzstan N = 306		Greece N = 90		Total N = 1040		Total without Kyrgyzstan N = 734	
	B	95%CI	B	95%CI	B	95%CI	B	95%CI	B	95%CI	B	95%CI
Male	1.50	(-5.02–8.01)	-4.01	(-10.65–2.63)	2.17	(-4.50–8.84)	-.91	(-14.07–12.24)	-0.17	(-3.83–3.50)	-1.17	(-5.56–3.21)
Age (years)	-.02	(-.27–.23)	.00	(-.18–.19)	0.09	(-0.15–.33)	.48	(-.03–.99)	0.05	(-0.08–0.17)	0.03	(-0.11–0.17)
Higher education†	3.26	(-2.97–9.49)	-2.26	(-7.16–2.64)	-7.09	(-18.38–4.21)	-3.40	(-29.30–22.49)	-0.92	(-4.54–2.69)	-0.22	(-4.03–3.59)
Working	-5.55	(-11.33–.22)	-4.98	(-10.03–.06)	-3.26	(-8.98–2.47)	-6.73	(-20.45–6.99)	-4.73*	(-7.82– -1.65)	-5.34*	(-9.00– -1.67)
Having children	3.11	(-5.30–11.52)	2.74	(-4.50–9.98)	19.50	(-1.63–40.63)	-5.24	(-22.24–11.75)	3.13	(-1.94–8.20)	2.13	(-3.09–7.35)
Ever smoker	1.69	(-13.17–16.55)	8.34*	(1.57–15.12)	5.42	(-1.05–11.89)	2.42	(-11.25–16.08)	5.97*	(1.73–10.22)	6.39*	(0.77–12.01)
Solid fuel use	.53	(-24.18–25.25)	5.73*	(.90–10.56)	2.43	(-2.91–7.76)	1.74	(-9.21–12.69)	3.94*	(0.56–7.31)	4.94*	(0.59–9.29)
Severity in MRC-scale	9.15*	(4.50–13.79)	8.42*	(6.38–10.46)	8.44*	(5.81–11.07)	12.62 *	(8.04–17.19)	8.92*	(7.47–10.36)	9.12*	(7.39–10.33)
Comorbidity, at least 1	11.35*	(3.70–19.01)	-1.92	(-6.26–2.42)	1.61	(-4.33–7.56)	4.04	(-6.53–14.61)	3.15	(-2.62–8.93)	4.09	(-4.79–12.98)

*B = unstandardized coefficients. CI confidence interval. MRC = medical research council breathlessness scale. *Value was considered statistically significant. Missing values excluded pairwise: N (%) for education 1 (0.1) for Uganda, working status 1 (0.1) for Vietnam, solid fuel use 3 (0.3) for Uganda, Kyrgyzstan and Greece, MRC-score 1 (0.1) for Uganda.*
